# Serum Levels of Fetal Antigen 1 in Extreme Nutritional States

**DOI:** 10.5402/2012/592648

**Published:** 2012-07-15

**Authors:** Alin Andries, Andreas Niemeier, Rene K. Støving, Basem M. Abdallah, Anna-Maria Wolf, Kirsten Hørder, Moustapha Kassem

**Affiliations:** ^1^KMEB laboratory, Department of Endocrinology and Center for Eating Disorders, Odense University Hospital, 5000 Odense, Denmark; ^2^Department of Orthopedics and Department of Biochemistry and Molecular Cell Biology, University Medical Center Hamburg-Eppendorf, 20246 Hamburg, Germany; ^3^Department for General, Visceral, and Transplantation Surgery, University Hospital Ulm, 89069 Ulm, Germany; ^4^Stem Cell Unit, Department of Anatomy, College of Medicine, King Saud University, Riyadh 11461, Saudi Arabia

## Abstract

*Objective*. Recent data suggest that fetal antigen (FA1) is linked to disorders of body weight. Thus, we measured FA1 serum levels in two extreme nutritional states of morbid obesity (MO) and anorexia nervosa (AN) and monitored its response to weight changes. *Design*. FA1 and insulin serum concentrations were assessed in a cross-sectional study design at defined time points after gastric restrictive surgery for 25 MO patients and 15 women with AN. *Results*. Absolute FA1 serum levels were within the assay normal range and were not different between the groups at baseline. However, the ratio of FA1/BMI was significantly higher in AN. FA1 was inversely correlated with BMI before and after weight change in AN, but not in MO patients. In addition, MO patients displayed a significant concomitant decrease of FA1 and insulin with the first 25% of EWL, while in AN patients a significant increase of FA1 was observed in association with weight gain. *Conclusion*. FA1 is a sensitive indicator of metabolic adaptation during weight change. While FA1 serum levels in humans generally do not correlate with BMI, our results suggest that changes in FA1 serum levels reflect changes in adipose tissue turnover.

## 1. Introduction

Fetal antigen 1 (FA1), also known as preadipocyte factor 1 (pref-1) or Delta-like 1 (Dlk1), was originally isolated from amniotic fluid [1]. It is a member of the epidermal growth factor (EGF) superfamily. FA1 refers to the soluble form of the protein which is released into the circulation and body fluids after proteolytic cleavage of the larger membrane-bound Dlk1 [2]. FA1 is a growth and differentiation factor widely expressed during fetal development and is progressively downregulated after birth [3]. In adults, FA1 is expressed in neuroendocrine tissues, for example insulin producing beta cells of the islets of Langerhans, growth hormone producing cells in the pituitary gland [4], adrenal glands, and sex hormone-producing cells of the gonads [3, 5], as well as specific areas in the central nervous system [6]. FA1 is known to regulate several cell differentiation pathways, for example, adipogenesis [7], neuroendocrine differentiation [3], hepatocyte differentiation [8], hematopoiesis [3], and osteogenesis [9, 10]. FA1 can be measured in serum and body fluids using ELISA assay, and its levels do not differ between males and females. Also, serum FA1 does not exhibit age-related changes beyond adolescence, diurnal variation, or variations during the menstrual cycle [11, 12].

 Recent data from genetically modified mouse models suggest a role of FA1 in adipose tissue turnover. Dlk1-deficient mice exhibit an obese phenotype associated with changes in serum lipids [13], while Dlk1-overexpressing mice with high serum levels of FA1 display a decreased fat mass, a phenotype reminiscent of lipodystrophy, decreased glucose tolerance, and insulin resistance [14, 15]. Also, recent mouse studies suggested that Pref-1/FA1 is a factor which may influence the susceptibility to the metabolic syndrome. FA1 inhibited adipogenesis and prevented diet-induced obesity [16]. In humans, the relationship between FA1 serum levels and total body mass or fat mass is less clear. In the human syndrome of maternal uniparental disomy (matUPD14) where Dlk1/Pref-1 is silent, the patients exhibit a number of developmental abnormalities including obesity, hypotonia, premature puberty, macrocephaly, short stature, and small hands[17, 18].

In order to further elucidate the relationship between circulating FA1 levels and changes in body weight in humans, we studied two extreme nutritional states, morbid obesity (MO) and anorexia nervosa (AN), before and after weight change.

The WHO defines a body-mass index (BMI) above 40 kg/m^2^ as class III obesity (or morbid obesity (MO)) and bariatric surgery is the only effective treatment option for ensuring weight loss and significant improvement in the metabolic state [19–22]. On the other extreme of pathological nutritional states, AN is a psychiatric disorder primarily characterized by a self-induced weight loss under the WHO defined lowest normal BMI of 18.5 kg/m^2^. Patients with AN exhibit complex endocrine and metabolic alterations occurring in AN in response to undernourishment and starvation [23].

Little is known about serum levels of FA1 in these extreme nutritional states of MO and AN. Two cross-sectional studies have been published on the subject, reporting elevated FA1 serum levels in both MO patients [24] and AN patients [25] as compared to healthy controls. Thus, the goals of the current study are to (a) asses FA1 levels of MO and AN patients and (b) analyze changes of FA1 levels in response to weight loss of MO and weight gain of AN patients. As FA1 is produced in pancreatic beta cells, we also investigated if there was a correlation between FA1 and changes in insulin levels under these nutritional conditions.

## 2. Methods

### 2.1. Obese Subjects

Twenty-five obese subjects, 17 women and 8 men, with a median age of 40 (range 24–56) and median BMI of 48 kg/m^2^ (range 41–70 kg/m^2^) underwent bariatric surgery at the Evangelische Krankenhaus Dinslaken, Germany. To qualify for the study, patients fulfilled the criteria established by the American Society for Bariatric Surgery [26]. The bariatric surgery (gastroplasty) was performed according to the standards for laparoscopic and open surgical treatment of morbid obesity. In all the MO patients, anthropometrics and metabolic indices were measured preoperatively and at defined points of excess weight loss (EWL) of 25%, 50%, and 75% after surgery. FA1 was measured in all patients before and in 21 patients after bariatric surgery. Preoperatively, the obese patients were fasting, while at the follow-up consultations patients did not strictly adhere to this recommendation.

### 2.2. AN Patients

Fifteen women with AN were studied at the specialized unit for Eating Disorders at Odense University Hospital, Denmark. The diagnosis of AN was based on the criteria from DSM-IV [27]. Mean age was 23.4 years (range 16–41 years). Median BMI was 15.30 kg/m^2^ (range 12.0–17.0 kg/m^2^). All patients were outpatients. None of the patients suffered from primary endocrine diseases, nor were they taking any medication known to influence fat metabolism or the nutritional state. All patients were amenorrheic and none were taking oral contraceptives. The local scientific ethical committee approved the study. All participants signed written informed consent.

In the AN group, body weight and height were measured and BMI was calculated at the study start and after 2–6 months in all subjects. The BMI changes were rather small (median increase of 3.45% from baseline) but statistically significant (*P* < 0.01) corresponding to an approximate weight gain of more than 10%. Serum FA1 and insulin were measured fasting at the same time of day at both baseline and followup. FA1 was measured in all 15 AN patients. In the AN group fasting blood glucose and insulin were measured for 11 out of 15 patients before weight change and 10 patients after weight change.

### 2.3. Biochemical Analysis

Serum FA1 was quantified using a sandwich ELISA employing polyclonal anti-FA1 antibodies purified by immunospecific affinity chromatography. The method and assay parameters have previously been described in detail [12]. The reference interval for serum (s)-FA1 was 12.3–46.6 ng/mL (age 19–60 years) [12]. Glucose was determined by the glucose dehydrogenase method (Merck, Darmstadt, Germany). Insulin was analyzed by microparticle enzyme immunoassay in the MO subjects, whereas in the AN patients it was determined by a double-antibody RIA (Kabi, Pharmacia Diagnostics, Uppsala, Sweden).

### 2.4. Statistics

The statistical analyses were performed using SPSS version 15.0 (SPSS, Chicago, IL, USA) and STATA/IC 12.0 (StataCorp, College Station, TX, USA). Comparisons between groups were performed using the Mann-Whitney *U* test. Mean ± SD and paired *t*-test were performed for testing statistical *t*-changes within the same group. A natural logarithmic transformation was performed for variables with non-normal distribution. Bivariate correlations were estimated using Pearson and Spearman's coefficients. Statistical significance in all analyses was *P* < 0.05.

## 3. Results

**Figure 1:**
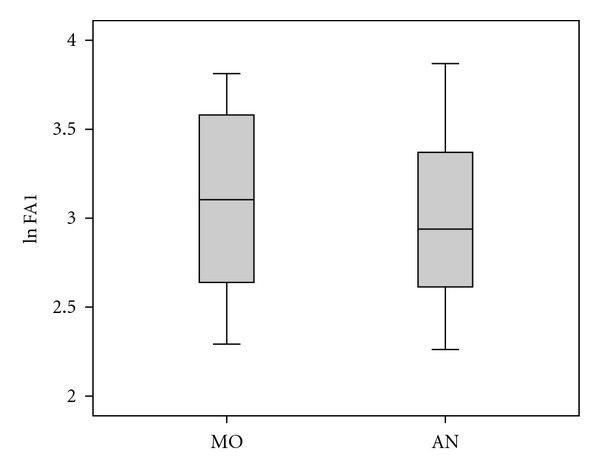
Similar FA1 levels in MO and AN at baseline. Serum levels of FA1 were similar at baseline in both MO (*n* = 25) and AN (*n* = 15) patients. The error bars represent standard deviations and confidence limits.

**Figure 2:**
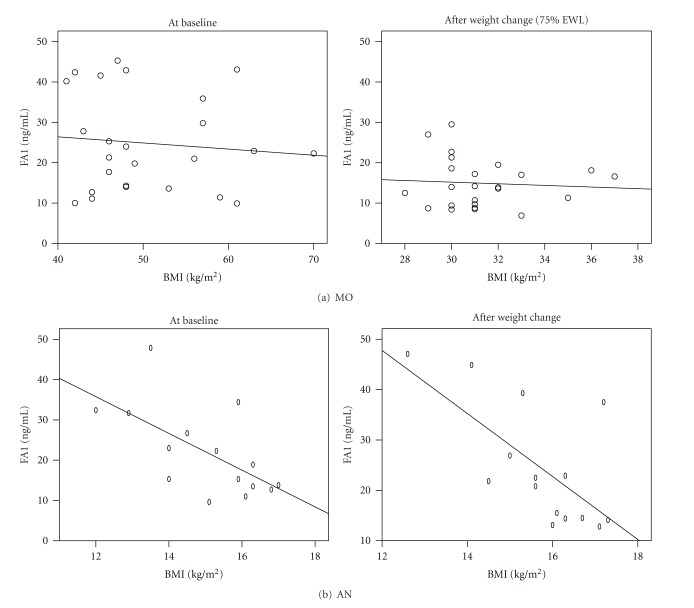
FA1 correlates with BMI in AN but not in MO. (a) We found no significant correlation between FA1 and BMI in MO, neither at baseline nor after weight change. (b) In AN, we found that FA1 slightly correlated to BMI both before (FA1 = 90.5−4.6 ∗ BMI, *R*-squared = 0.4; *P* = 0.012) and after (FA1 = 122.9−6.3 ∗ BMI, *R*-squared = 0.45; *P* = 0.006) weight change.

The baseline anthropometric and biochemical characteristics of the patients are shown in Table 1. We found no significant differences regarding FA1 levels in the two subgroups at baseline (Table 1 and Figure 1). While FA1 did not correlate with BMI at individual time points, we observed a slight inverse correlation with BMI both before (*r* = 0.4; *P* = 0.012) and after (*r* = 0.45; *P* = 0.006) weight change in the AN subgroup (Figure 2).

**Figure 3:**
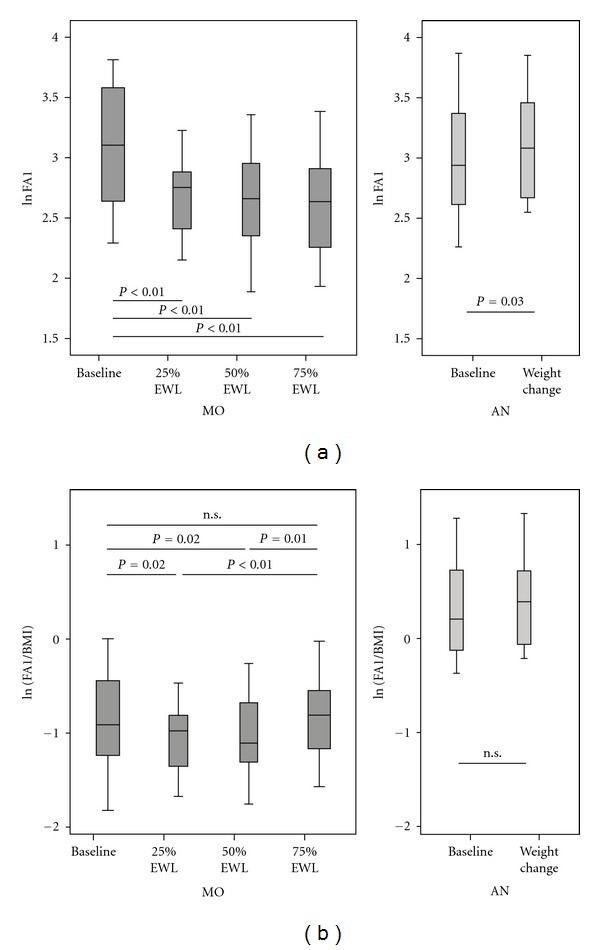
(a) FA1 levels decrease with weight loss in MO and increase with weight gain in AN. FA1 levels changed significantly both during and after weight change. The error bars represent standard deviations and confidence limits. (b) The FA1/BMI ratio is lower in MO and remains constant upon weight change. In MO, FA1/BMI ratio was lower than in AN. It decreased significantly at 25% EWL, followed by a slow ascending course to the initial levels. The error bars represent standard deviations and confidence limits.

Upon weight loss in MO patients, FA1 decreased significantly at all time points of EWL as compared to baseline. On the other hand, we observed a significant increase in FA1 levels in AN patients upon weight gain.  Figure 3(a) shows the FA1 serum concentrations in MO and AN patients before and after weight change.

In order to estimate the contribution of fat tissue to the serum level of FA1, we calculated the amount of FA1 expressed per BMI unit. The FA1/BMI ratio was significantly lower in the MO group (Figure 3(b)). In addition, we observed that FA1/BMI ratio did not change significantly between before and after weight change in the two subgroups (Figure 3(b)), while in MO it changed significantly at intermediary EWL points. We also observed a significant, parallel drop in both FA1 and insulin in MO during weight loss (data not shown), while in AN insulin correlated significantly with FA1 only before weight change (*r* = 0.65; *P* = 0.031).

By fitting our data to a multiple linear regression model, we tested the effect of different covariates on the random variation of the serum level of FA1. Both BMI (*r* = 0.39, *P* < 0.001) and age (*r* = 0.34, *P* = 0.04) were found to have a significantly negative effect on FA1. Interestingly, we found that males seemed to have lower FA1 values than females with same age and BMI, but this effect was not above the chosen level of statistic significance (*P* = 0.09).

## 4. Discussion

In the present study, we examined the possible use of FA1 as a biomarker for changes in fat mass and investigated the dynamic changes of its serum levels during weight change in patients at two extreme nutritional states: MO and AN.

We did not detect any differences in serum levels of FA1 between MO and AN in our study, and their levels were within the normal range of our ELISA assay. Previous studies have shown that serum levels of FA1 in MO [24] and AN [25] were significantly higher compared to lean controls. In these studies, the absolute values of serum FA1 levels were within the normal range of our assay (12.3–46.6 ng/mL) [12], but the assays employed in these studies were different, thus not directly comparable. The absence of significant changes in serum FA1 levels between MO and AN is at variance with our findings in rodent models. Our group has previously studied the effects of circulating FA1 on fat mass *in vivo* in mice with high serum levels of FA1 (198 ± 74 ng/mL) [10] and found that increased serum level of FA1 was associated with a significant reduction of total body weight and fat mass. Also, dlk1/FA1-overexpressing mice exhibited decreased fat mass [14, 15]. Our results confirm these findings in humans, showing that the serum levels of FA1 were significantly inversely correlated with BMI, independently of the individual nutritional status. The available data in humans, therefore, suggest that steady state levels of serum FA1 are not a sensitive biomarker for the nutritional state of an individual.

The serum level of several adipokines (e.g., leptin) correlates with fat mass. FA1 is produced by adipocyte precursors and its expression is downregulated in mature adipocytes [10]. Thus, we expressed FA1 per BMI in order to estimate the contribution of fat tissue to FA1 serum levels. We found that the ratio of FA1/BMI in MO was significantly lower than in the AN group and it changed significantly during weight loss, suggesting that newly recruited adipocyte precursors contribute to serum pool of FA1. The presence of an inverse correlation between FA1 and BMI in AN, but not in MO patients, suggests that fat tissue undergoes a higher degree of turnover in AN than in MO. Our findings that a minor weight gain of 10% in the AN group was accompanied by a significant increase in serum FA1 levels, while in the MO group a significant fall in FA1 levels occurred after bariatric surgery with 25% EW, corroborate this hypothesis and provides evidence that changes in FA1 levels in the serum reflect change in fat tissue mass.

A significant correlation between baseline serum levels of FA1 and insulin levels was found the AN group and not in the MO. However, the absence of the correlation in the MO group may have been caused by the inconsistency in obtaining fasting samples in this group. It is not clear whether changes in serum FA1 was caused by changes in insulin levels since FA1 is produced in pancreatic beta cells [28]. In contrast with previous reports, we found that serum FA1 levels are inversely correlated to age and might be influenced by sex, sustaining our previously reported reports on the possible regulation of serum FA1 by systemic hormones, for example estrogen [29] and growth hormone [10]. However, further studies are needed to confirm the relationship between insulin production and serum levels of FA1.

## 5. Conclusion

In conclusion, steady state serum FA1 in human adults is not a biomarker for fat mass but it can be employed to monitor acute changes in fat mass. We propose that these changes in serum FA1 are caused by changes in the pool of FA1-producing adipocyte precursor cells that are recruited during periods of acute turnover of adipose tissue, a hypothesis which will have to be confirmed by other studies.

## Figures and Tables

**Table 1:** Anthropometric and biochemical characteristics at baseline.

	Overall (*n* = 40)	Obese (*n* = 25)	AN (*n* = 15)
Age (years)*	33.52 (16–56)	40 (24–56)	23.4 (16–41)
Gender	23♀/17♂	8♀/17♂	15♂
BMI (kg/m^2^)*	37.24 (12–70)	47.2 (41–70)	15.30 (12–17)
Insulin (*μ*U/mL)*	10.18 (1–35)	13 (1–35)	3.58 (1.5–7.17)^¶^
FA1 (ng/mL)	23.72 (9.6–47.9)	22.3 (9.9–45.3)	18.9 (9.6–47.9)

Numbers indicate median (min-max),

^¶^ *n* = 10;

*P < 0.001.

## Abbreviations

FA1: fetal antigen,
pref-1: preadipocyte factor,
Dlk1: delta-like preadipocyte factor,
EGF: epidermal growth factor,
AN: anorexia nervosa,
MO: morbid obesity,
EWL: excess weight loss,
BMI: body mass index,
WHO: World Health Organization,
DM: diabetes mellitus,
SOS: Swedish Obese Subjects study.
